# Palmoplantar Maculopapular Rash Mimicking Secondary Syphilis in Infectious Mononucleosis After Sequential Antibiotic Exposure: A Case Report

**DOI:** 10.7759/cureus.118579

**Published:** 2026-10-25

**Authors:** Emily Smith, Victoria Rodriguez Bajo, Soumitra Roy

**Affiliations:** 1 Internal Medicine, Dartford and Gravesham NHS Trust, Dartford, GBR

**Keywords:** drug hypersensitivity reactions, epstein-barr virus infection, infectious mononucleosis, maculopapular rash, penicillin allergy

## Abstract

Infectious mononucleosis caused by Epstein-Barr virus (EBV) commonly presents with pharyngitis, lymphadenopathy, and fever. Cutaneous eruptions may occur, particularly following antibiotic exposure. We report a case of a man in his late 20s who developed a widespread maculopapular rash following sequential exposure to multiple antibiotics in the setting of acute EBV infection. The rash progressed to involve the palms and soles, raising concern for secondary syphilis and a possible drug hypersensitivity reaction. Investigations demonstrated marked lymphocytosis, elevated inflammatory markers and deranged liver enzymes. Throat culture was negative for beta-haemolytic streptococci and the antistreptolysin O titre did not suggest recent streptococcal infection. *Treponema pallidum* antibody testing and measles polymerase chain reaction (PCR) were negative. Subsequent EBV PCR and repeat serology supported acute EBV infection. The patient improved with conservative management, with resolution of the rash and normalization of the white cell count and liver enzymes at follow-up. Retrospective review also identified prior intravenous amoxicillin exposure during admission for appendicitis, adding further uncertainty regarding the cause of the eruption. This case highlights the diagnostic complexity of rash occurring during acute EBV infection following multiple antibiotic exposures and the importance of considering both EBV-associated exanthem and drug hypersensitivity in the differential diagnosis. Accurate assessment of suspected penicillin allergy is important to guide future antibiotic prescribing.

## Introduction

Infectious mononucleosis, most commonly caused by Epstein-Barr virus (EBV), typically presents with fever, cervical lymphadenopathy and pharyngitis, often accompanied by tonsillar enlargement and exudate [1]. Lymphocytosis and abnormalities in liver function tests are also common laboratory findings. Cutaneous manifestations may occur, classically presenting as a diffuse maculopapular eruption, particularly following treatment with aminopenicillins, such as amoxicillin, for presumed streptococcal pharyngitis [2]. However, the frequency and underlying mechanism of this association remain debated. 

Historically, aminopenicillin-associated rash was considered a characteristic feature of infectious mononucleosis. However, recent evidence suggests that this association may be less frequent than previously reported. A recent systematic review and meta-analysis estimated that rash occurred in 43% of patients with infectious mononucleosis following aminopenicillin exposure, compared with 15% following other antibiotics and 14% in the absence of antibiotic treatment [2]. These findings suggest that the development of rash cannot be attributed solely to a simple virus-drug interaction, with both virus-associated immune dysregulation and genuine aminopenicillin sensitization proposed as possible mechanisms [3,4]. An EBV-associated exanthem and genuine antibiotic hypersensitivity likely represent distinct, but potentially overlapping, explanations for an eruption. 

The distribution and characteristics of an eruption may provide important diagnostic clues. Although not diagnostically specific, palmoplantar involvement may raise suspicion for syphilis as a recognised feature of the disease in its second phase, during which patients may also present with fever, pharyngitis and lymphadenopathy [5]. Furthermore, compared with the typical EBV-associated exanthem, antibiotic-associated eruptions are more likely to be pruritic and involve the palms and soles [6]. 

Our patient presented following appendicectomy with a widespread, non-pruritic rash involving the palms and soles after exposure to multiple antibiotics. This presentation created diagnostic uncertainty between an EBV-associated exanthem, antibiotic-related hypersensitivity and secondary syphilis. Retrospective medication reconciliation also identified preceding exposure to intravenous amoxicillin during admission for presumed acute appendicitis. The case highlights the difficulty of attributing cutaneous eruptions in patients with infectious mononucleosis when multiple sequential antibiotics have been administered complicating allergy attribution. It also emphasizes the importance of considering alternative infectious diagnoses, particularly secondary syphilis, when the distribution or clinical characteristics of the eruption are atypical for EBV-associated exanthem. 

## Case presentation

A man in his late 20s presented to the Accident and Emergency (A&E) Department with a three-day history of a non-pruritic, generalised, blanching maculopapular rash. Ten days previously, he had attended the A&E Department with abdominal pain and subsequently underwent a same-day emergency laparoscopic appendicectomy following computed tomography findings suggestive of appendicitis. An inflamed appendix was noted intraoperatively. The patient reported a sore throat one day prior to surgery, which persisted postoperatively. Consequently, three days after surgery, he was prescribed a five-day course of oral phenoxymethylpenicillin 500 mg four times a day by his general practitioner (GP). After four days of treatment with phenoxymethylpenicillin, the patient developed a maculopapular rash that started on the face before spreading to the trunk, arms and groins. The antibiotic was subsequently changed to oral erythromycin 500mg four times a day; however, he re-presented to the A&E Department the following day due to worsening of the generalised rash. 

His medical history included asthma, hay fever and allergies to cats and dogs, and vaccinations were reported to be up to date. He reported no recent travel to tropical or endemic areas. Two months prior to presentation, whilst abroad, he developed dysuria and penile discharge and was treated empirically with unknown antibiotics for a presumed urinary tract infection. He reported no new sexual partners or known sexual exposures during this period and was in a regular sexual relationship. 

On admission, the patient reported throat pain on swallowing. He denied fever, chills, headache, dyspnea or respiratory compromise. His observations were: heart rate 90 beats per minute (reference range: 60-100 beats/minute), blood pressure 124/86 mmHg (reference range: 90/60mmHg to 120/80mmHg), respiratory rate 18 breaths per minute (reference range: 12-20 breaths/minute), oxygen saturation 96% on room air (normal range: 95-100%)and he was afebrile. On examination, the rash had progressed to involve the palms (Figure 1A) and soles, although oral mucosa was spared.

**Figure 1:**
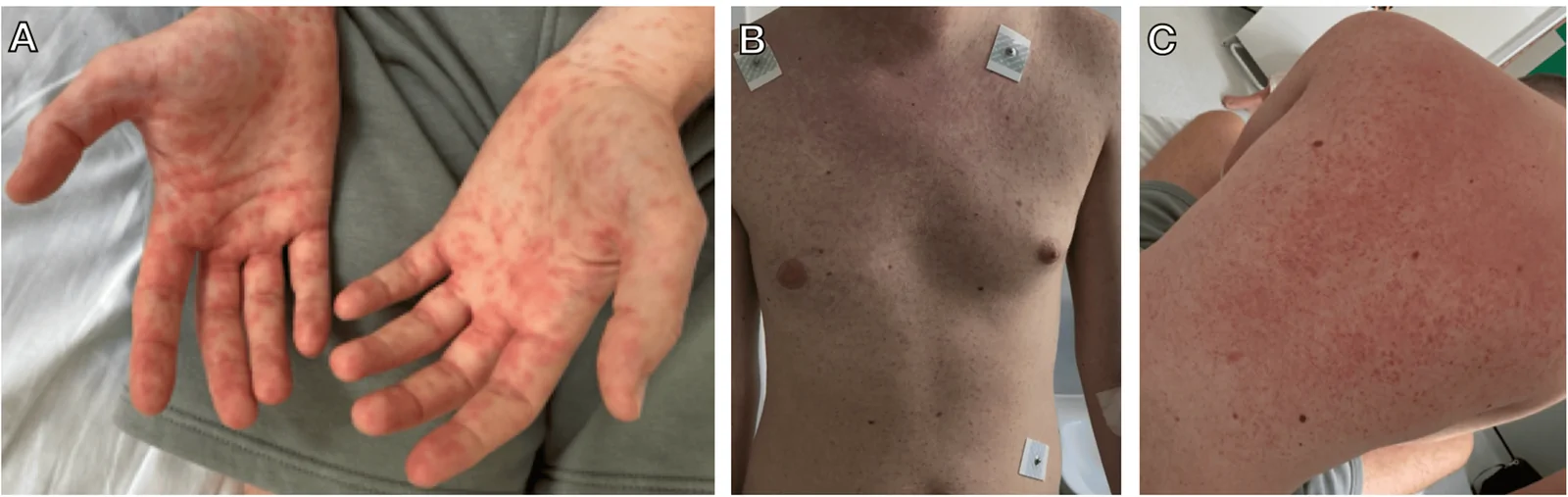
Generalised maculopapular rash Affecting the palms (A), anterior torso (B) and posterior torso (C).

There was bilateral tonsillar enlargement with white tonsillar exudate. There was no evidence of a peritonsillar abscess. Cervical lymphadenopathy was present. Cardiovascular examination demonstrated normal heart sounds with no murmurs. The lung fields were clear on auscultation. The abdomen was soft and non-tender, with mild splenomegaly and normal bowel sounds. 

Initial blood investigations demonstrated an elevated white cell count (WBC) with lymphocytosis and elevated C-reactive protein (CRP). Eosinophil count was within normal range. Liver function tests were deranged, with elevated alkaline phosphatase (ALP) and alanine aminotransferase (ALT) (Table 1). 

**Table 1:** Laboratory results during admission and at follow-up Days are counted from admission; - denotes result not available.

Investigation	Reference range	On admission (Day 0)	Repeat (Day 5)	GP follow-up (Day 11)
White cell count	3.6–11.0 × 10⁹/L	17.5 × 10⁹/L	10.6 × 10⁹/L	5.7 × 10⁹/L
Lymphocytes	1.0–4.0 × 10⁹/L	12.8 × 10⁹/L	6.7 × 10⁹/L	3.2 × 10⁹/L
Eosinophil count	0.0–0.4 × 10⁹/L	0.2 × 10⁹/L	0.2 × 10⁹/L	-
C-reactive protein	<5 mg/L	45.8 mg/L	14.2 mg/L	-
Alkaline phosphatase	30–130 U/L	158 U/L	101 U/L	88 U/L
Alanine aminotransferase	7–56 U/L	166 U/L	85 U/L	55 U/L

Antibiotics were discontinued on day 0 of admission due to concern regarding a possible antibiotic-associated allergic reaction and the increasing suspicion of a viral illness. The patient was managed conservatively with analgesia. 

Initial EBV serology performed in the community (five days prior to admission with rash) was inconclusive, with undetectable EBV nuclear antigen (EBNA) IgG and equivocal EBV IgM. An acute EBV infection could not be established at this point. Human immunodeficiency virus (HIV) screening (on day 0 of admission) via fourth-generation antigen/antibody immunoassay was non-reactive. Throat culture (sampled on day one of admission) did not isolate beta-haemolytic streptococci, and an antistreptolysin O (ASO) titre (sampled on day four of admission) of <200 IU/mL did not suggest recent streptococcal infection. Measles serology (sampled on day three of admission) demonstrated positive IgG, consistent with previous immunity from vaccination, while measles polymerase chain reaction (PCR) testing was negative. Treponema pallidum antibodies (sampled on day four of admission) were not detected. Subsequent viral PCR (sampled on day four of admission) detected EBV DNA with 52,100 copies/mL, while cytomegalovirus (CMV) DNA was not detected. Repeat EBV serology (sampled on day four of admission) demonstrated equivocal EBNA IgG and positive EBV IgM, suggestive of acute EBV infection. 

Based on the clinical presentation and subsequent EBV PCR and serological findings, a diagnosis of infectious mononucleosis was made. The patient was managed conservatively and discharged once oral intake was adequate on day six of inpatient admission. He was safety-netted and advised to avoid contact sports due to the risk of splenic rupture. 

At GP follow-up on day 11 after admission with rash, the patient reported significant clinical improvement, with resolution of the rash. Repeat blood tests demonstrated normalization of the WBC and liver enzymes. His allergy status was updated to indicate a penicillin allergy; however, further allergy testing has not yet been performed.

On further review of his medical records, it was noted that the patient had received two days of intravenous amoxicillin 1g three times a day during his admission for appendicitis and laparoscopic appendicectomy. 

## Discussion

This case highlights the diagnostic uncertainty that can arise when a young adult presents with a widespread maculopapular rash following multiple antibiotic exposures. Palmoplantar involvement raised concern for secondary syphilis, whilst differentials also included an EBV-associated viral exanthem, an antibiotic-associated eruption or a true penicillin allergy. Attribution of the rash was further complicated by the multiple sequential antibiotic exposures, which made it difficult to determine the contribution of each antibiotic, if any. In addition, the preceding acute appendicitis may have represented an early manifestation of EBV infection. 

Progression of the rash to involve the palms and soles added diagnostic complexity. Palmoplantar involvement is classically associated with secondary syphilis [5] and therefore appropriately prompted investigation in our patient. Nevertheless, EBV infection can also produce atypical cutaneous manifestations which mimic secondary syphilis. Other EBV-associated mimics have also been reported. One such report describes EBV-associated infectious mononucleosis in a seven-year-old with a rash mimicking Kawasaki disease [7]. As in our case, amoxicillin had been administered shortly before the onset of the eruption, although it had been prescribed for otitis media rather than suspected streptococcal pharyngitis. This report supports the importance of considering EBV within the differential diagnosis for an eruption occurring alongside pharyngitis and systemic features suggestive of infectious mononucleosis. 

The pathophysiological mechanism of antibiotic-associated rash during EBV infection remains incompletely understood. One proposed mechanism is a transient virus-mediated alteration of the immune response, resulting in loss of tolerance and a reversible delayed-type hypersensitivity reaction to the antibiotic [3]. However, there is evidence that some patients with infectious mononucleosis who develop rash following amoxicillin may develop a genuine drug-specific sensitization rather than a purely virus-mediated eruption. In a small study, Renn et al. demonstrated drug-specific lymphocyte reactivity to amoxicillin in three of four patients, with findings supported by in vivo skin testing, suggesting that true drug sensitization may occur in a subset of patients [4]. A subsequent study has continued to explore the possibility of true penicillin sensitization in this setting [8]. 

Retrospective medication reconciliation identified exposure to intravenous amoxicillin during admission for appendicitis, followed by phenoxymethylpenicillin and then erythromycin, making it difficult to attribute the eruption to any individual antibiotic, or to antibiotics at all. Although the initial amoxicillin exposure provides a plausible antecedent to subsequent sensitisation, the later phenoxymethylpenicillin exposure occurred closer to rash onset, while the eruption also developed in the context of confirmed acute EBV infection. The non-pruritic nature of the eruption was compatible with a viral exanthem, although its palmoplantar involvement was atypical and raised the possibility of an antibiotic-associated eruption as well as secondary syphilis. The available evidence therefore does not allow the rash to be confidently attributed to EBV infection or to a specific antibiotic. 

This uncertainty has clinical implications. Inaccurate penicillin allergy labelling can result in the use of alternative antibiotics, suboptimal antimicrobial therapy and adverse antimicrobial stewardship outcomes [9]. Conversely, attributing a rash solely to an EBV-associated antibiotic eruption and failing to recognise a genuine drug hypersensitivity reaction may put a patient at risk of a more severe reaction if re-exposed. In our case, distinguishing between these possibilities was particularly challenging given exposure to several antibiotics over a relatively short period. 

An additional consideration is whether the patient’s acute appendicitis represented a complication of the underlying EBV infection. Although uncommon, acute appendicitis has been reported as a rare initial manifestation of infectious mononucleosis, including without preceding upper respiratory symptoms [10]. One proposed mechanism includes obstruction of the appendiceal lumen due to EBV-associated lymphoid tissue swelling, resulting in acute appendicitis [11]. In our patient, the sore throat preceding appendicitis, followed by lymphocytosis, elevated liver enzymes and confirmed acute EBV infection, raises the possibility that the appendicitis was related to EBV infection. However, given the absence of contemporaneous EBV testing or histopathological evidence, a causal relationship cannot be established. 

There are several strengths to this case. The patient had a detailed clinical and laboratory work-up, including investigation for streptococcal infection, measles, CMV and secondary syphilis, making several important differential diagnoses less likely. Serial blood results also demonstrated resolution of the lymphocytosis and liver function abnormalities, with an improving CRP alongside clinical improvement, supporting a self-limiting illness. In addition, retrospective medication review provided important additional information when interpreting the rash. Nonetheless, the main limitation of this case was incomplete allergy assessment. Therefore, although EBV infection provides a strong explanation for the clinical presentation, uncertainty remains whether the rash represented an EBV-associated viral exanthem, a delayed hypersensitivity reaction to one of the antibiotics, or a combination of both. This limitation is important when considering the patient’s future antibiotic prescribing. 

## Conclusions

This case has implications for everyday clinical practice. Firstly, EBV infection should remain within the differential diagnosis for a patient presenting with pharyngitis and maculopapular rash, particularly when lymphocytosis and deranged liver function tests are present. Secondly, palmoplantar involvement should prompt consideration of secondary syphilis but should not exclude alternative viral causes. Thirdly, an accurate antibiotic history, including retrospective medication reconciliation, is essential when assessing suspected antibiotic allergy. Finally, acute appendicitis may rarely occur in association with infectious mononucleosis and may precede recognition of the underlying infection. 

Overall, this case illustrates the diagnostic complexity of an atypical rash in the context of EBV infection and sequential antibiotic exposure. The preceding appendicitis raises the possibility of an association with EBV, although this remains unproven, while the palmoplantar eruption mimicked secondary syphilis. The case also highlights the ongoing challenge of distinguishing infection-associated exanthems from genuine antibiotic hypersensitivity. With emerging evidence suggesting that true drug sensitization may occur in some patients, rather than the reaction being universally attributable to EBV alone, further investigation of suspected antibiotic allergy is important. Where clinical circumstances permit, accurate identification of penicillin allergy status may prevent both unnecessary avoidance of first-line antibiotics and, equally importantly, inadvertent re-exposure in patients with true hypersensitivity. 
